# Use of prior odds for missing persons identifications

**DOI:** 10.1186/2041-2223-2-15

**Published:** 2011-06-27

**Authors:** Bruce Budowle, Jianye Ge, Ranajit Chakraborty, Harrell Gill-King

**Affiliations:** 1 Institute of Investigative Genetics, 3500 Camp Bowie Blvd, University of North Texas Health Science Center, Fort Worth, TX, 76102, USA; 2 Department of Forensic and Investigative Genetics, 3500 Camp Bowie Blvd, University of North Texas Health Science Center, Fort Worth, TX, 76102, USA; 3 Department of Biological Sciences, Biology Building 210, P.O. Box 305220, University of North Texas, Denton, TX, 76203, USA

## Abstract

Identification of missing persons from mass disasters is based on evaluation of a number of variables and observations regarding the combination of features derived from these variables. DNA typing now is playing a more prominent role in the identification of human remains, and particularly so for highly decomposed and fragmented remains. The strength of genetic associations, by either direct or kinship analyses, is often quantified by calculating a likelihood ratio. The likelihood ratio can be multiplied by prior odds based on nongenetic evidence to calculate the posterior odds, that is, by applying Bayes' Theorem, to arrive at a probability of identity. For the identification of human remains, the path creating the set and intersection of variables that contribute to the prior odds needs to be appreciated and well defined. Other than considering the total number of missing persons, the forensic DNA community has been silent on specifying the elements of prior odds computations. The variables include the number of missing individuals, eyewitness accounts, anthropological features, demographics and other identifying characteristics. The assumptions, supporting data and reasoning that are used to establish a prior probability that will be combined with the genetic data need to be considered and justified. Otherwise, data may be unintentionally or intentionally manipulated to achieve a probability of identity that cannot be supported and can thus misrepresent the uncertainty with associations. The forensic DNA community needs to develop guidelines for objectively computing prior odds.

## Background

Mass disasters can result from natural, accidental and intentional causes. One of the tragedies of such events is loss of lives. Many jurisdictions seek to identify the victims so they can be returned to their respective families, for investigative purposes and/or for legal reasons (for example, resolution of estates, probate and criminal investigations). As in other forensic applications, identification is a matter of evaluating variables to reduce the pool of candidates with the intent of approaching individualization. Various characteristics or traits are used to assist in the identification of the human remains, including but not limited to skeletal features (for example, sex, age, stature and ancestry), dental comparisons, fingerprints, distinguishing marks (for example, tattoos and scars), medical devices and implants, other unique features, DNA profiles and, to a much lesser extent, eyewitness accounts and sometimes personal items. However, the frequency of the observed value of each variable needs to be assessed appropriately to effectively reduce the candidate pool.

### Current practice and possible limitations

For the past two decades, DNA typing has played a more prominent role in the identification of human remains, and particularly so for highly decomposed and fragmented remains [1-12]. DNA profiles from recovered unidentified human remains may be compared with direct reference samples (for example, toothbrush, razor and hairbrush) and/or profiles from relatives (that is, an indirect comparison or kinship analysis) to identify possible associations. The strength of the genetic associations is often quantified by calculating a likelihood ratio (LR). The LR is used to evaluate whether there is evidence to support a specified biological relationship or direct identification. The literature is replete with approaches to calculating the LRs for direct and kinship analyses [5,13-23] and therefore need not be discussed further herein.

The genetic evidence (that is, LR) can be combined with nongenetic evidence (summarized into a numerical value called "prior odds") to evaluate the probability of identity using Bayes' Theorem [24-28]. Bayes' Theorem is(1)

Thus, the prior odds figure into the ultimate posterior odds, and decisions are made on the basis of the posterior odds. Though the above translation of LR into posterior odds is simple, the multiplication of LR with prior odds implies that judgments or considerations used to set the prior odds must be independent of observations used in computing the LR. Consequently, the path creating the set and intersection of variables that contribute to the prior odds needs to be appreciated and well defined. Equally important is that the values used for the prior odds should be defensible. Assumptions regarding the frequency of the variables, especially without data, and the belief in the independence of variables may or may not be defensible. Serious thought and caution are warranted when providing prior odds or probability.

A typical scenario in mass disasters is that the prior probability is based on the known total number of missing persons or victims. In missing person cases with a pool of *v *victims, the prior probability is 1/*v*, and the prior odds for a specific missing person is 1/(*v *- 1), if no further information is provided and all missing persons are treated equally. The posterior odds is *p*/(1-*p*), where *p *is the posterior probability or probability of identity. Equation (1) can also be written as equation (2)(2)

A minimum threshold is set for an assertion regarding the probability of identity, for example, at 99.9% certainty for a posterior probability. The threshold is set by policy, but is a balance between maximizing identifications and minimizing false identifications [2,5,14]. Formally, the choice of a threshold for the posterior probability of identity is a matter of weighing the costs of false decisions. The requirement of a very high posterior probability (that is, > 99.9%) implies that obtaining false-positives is deemed worse than obtaining false-negatives. For given LR and *v *values, the distribution of the posterior probability (*P*) is shown in Table 1. For example, with 100 missing persons and a LR of 10^5 ^for a specific missing person association with a putative family, the posterior probability is greater than 99.9% certainty that the missing person belongs to the family. In general, for any fixed LR value, the higher the prior odds (for example, the lower the *v *value), the higher is the power of identification (that is, higher posterior odds). Thus, the limitations on reaching the prescribed threshold for probability of identity are low prior odds, the quality and quantity of DNA and, for kinship analyses, reference samples from informative relatives (the limitations of the latter two, in general, yield low LRs). In addition, the lack of a good chain of custody on antemortem biological samples can reduce the overall strength of the evidence.

**Table 1:** Distribution of the posterior probability (*P*) with a given LR and number of victims (*v*)

	Likelihood ratio (LR)				
	
Prior odds = 1/(*v *- 1)	100	1,000	10,000	100,000	1,000,000
1/(10-1)	0.917431	0.99108	0.999101	0.99991	0.999991
1/(50-1)	0.671141	0.953289	0.995124	0.99951	0.999951
1/(100-1)	0.502513	0.909918	0.990197	0.999011	0.999901
1/(500-1)	0.166945	0.667111	0.952472	0.995035	0.999501
1/(1,000-1)	0.090992	0.50025	0.909174	0.990109	0.999002
1/(2,000-1)	0.047642	0.333444	0.833403	0.980402	0.998005
1/(5,000-1)	0.019612	0.166694	0.666711	0.95239	0.995026

There has been some discussion in the literature about establishing the prior probability in mass disaster cases which recommends that it be based on the total number of unidentified victims and defining the population as open or closed [2,5,14]. For closed population cases, such as a plane crash, the number of victims (*v*) often is quantifiable. For open population cases, *v *is estimated and carries less certainty than for closed population cases, but is often manageable. As an example of setting a prior probability, in the aftermath of the attack on the World Trade Center (WTC), it was determined that a little less than 3,000 people died in the disaster [2]. The prior probability was set at approximately 1/3,000 [2] and could have been updated as identifications were made or increased by using gender [2]. Other nongenetic information often could not be included into the prior probability, because the vast majority of remains were severely fragmented.

Establishing the prior probability in cases of war or genocide is similar in principle to the WTC example, but there has been little guidance regarding the estimates. Indeed, while the forensic DNA community has made recommendations for using Bayes' Theorem, they have not addressed the variables that should be considered when establishing prior odds (beyond using the total number of missing persons) [29]. Because of a lack of definition, it is likely that some practices for estimating prior probabilities in such missing persons cases may not have been applied legitimately and thus at least warrant further discussion. For obvious reasons, we refrain herein from discussing cases still under deliberation; however, the following example from the literature illustrates the importance of objective specifications of prior odds.

Zupanič Pajnič *et al*. [30] used a prior probability of 1/89 for 88 missing victims of a post-World War II massacre in Slovenia. The number of victims was based on documents, but the authors suggested that some of the victims might reside in another site. Not all the remains could be identified. They did not consider the uncertainty that some of the victims other than the missing 88 might reside in the burial site. Thus, the prior probability of 1/89 might be too high.

Primarily, our discussion mainly involves considerations in formulating the prior odds (or prior probability) for missing persons identifications; these should be equally applicable to other scenarios as well. For example, some of the issues discussed herein have been raised in the context of forensic speaker recognition from voice recordings evidence data [31]. The intention is to raise awareness, and hence discussion, within the human identity community to address this gap that needs better-defined guidelines.

In instances of war or genocide, human remains typically are not found in one site but are distributed among multiple sites, manifests often are not available (and, if available, may not be entirely reliable) and bodies may have been moved. Anthropological data, eyewitness accounts and geography have been used to establish prior probability. Before the DNA evidence is analyzed, prior probability is established about the correctness of the hypothesis that the sample is from a particular victim. The prior probability should reflect reasonable beliefs about an event before receiving new information, such as the genetic evidence. Importantly, prior probability should be established before the genetic data are obtained, as the two values, prior odds and LR, are to be combined under the assumption of independence. Prior probabilities can be updated with new information. The simplest and most scientifically defensible approach is reducing the *v *value as identifications are made.

In mass grave situations, eyewitness accounts have been used by some to assert *a priori *that an individual was buried at a particular site. An anthropological assessment of the burial site remains determines that a minimum number of *n *individuals are at the burial site. The prior probability of that specific individual being at that site is then set at 1/*n*. As shown in Table 1, increasing the prior probability substantially from 1/*v *to 1/*n *can affect the posterior odds and, in some cases, can enable meeting a prescribed threshold. Thus, there is an incentive to reduce *v *to as small an *n *as possible. However, setting the prior probability at 1/*n *assumes that an eyewitness account is 100% accurate, an assumption that is hardly defensible. Scheck *et al*. [32] found that mistaken eyewitness accounts were a factor in 82% of wrongful convictions relying on eyewitness accounts, with the defendant subsequently being exonerated by DNA typing. Perceptions are distorted by the chaos and duress of the observational context and the individual limitations of the observer. However, such accounts might be considered if corroborated by mutually independent accounts. In situations where the DNA evidence demonstrates that a burial site population does not contain the reported individual, the prior probability of 1/*n *is rejected; such rejection of an association within a burial site in itself demonstrates a lack of 100% accuracy in an eyewitness account. Some lower prior probability should be selected that is bounded by 1/*v*, where *v *is the total number of missing persons.

In the above-described scenario, the prior probability clearly is a range between 1/*v *and 1/*n *(where *n *may be somewhat uncertain because of conditions within the site but where, for simplicity of argument, it is assumed to be correct). It might be more appropriate to provide a range to the fact finder or official responsible for assigning identity, as this would fairly convey that there is some degree of uncertainty. Forensic scientists tend to be reluctant to convey such uncertainty, either because the uncertainty is not appreciated [28,33] or because they believe the uncertainty will create confusion. A prior probability of 1/*v *is conservative and more defensible. Alternatively, the prior probability could be updated in the above-described scenario as 1/(*v-n*) [14]. Assuming that samples from each victim at the burial site yielded sufficient genetic information to eliminate the specific victim from being at that site, the prior probability could be bounded by the remaining number of unidentified victims. The adjustment of 1/(*v-n*) may seem trivial but in some scenarios could be substantial and, more importantly, can be scientifically defensible. Evaluation of the strength of the evidence can be more complicated because of variation in the structure of the reference families and should be considered (but is not discussed herein).

Data on the accuracy of eyewitness accounts are not available. Instead of DNA scientists and anthropologists providing the prior probability including an eyewitness account, the official who makes the decision about identity could be given the range of 1/*v *and 1/*n*, and he or she could apply his or her belief in the level of confidence of the eyewitness account. This approach prevents the scientist from providing a prior probability that is not readily estimated. However, it still does not obviate the need to collect data on the reliability of eyewitness accounts in chaotic or traumatic events; that is, corroboration, observer skills, credibility, independent accounts, how much accuracy decays over the time of the event and accounting of the incident, and so on. The official still would need the supporting data to select the best prior probability. The data to be collected are in some respects similar to the prior probabilities used in athlete doping [34]. Eyewitness data would likely be an average of witness accuracy, regardless of other influencing variables. A better estimate would be performance by the particular witness over multiple traumatic or challenging events. Unfortunately, the latter information cannot be practically obtained. Because of the number of variables that are likely to affect each witness in varying scenarios, a good prior probability for an eyewitness account might not be attainable and would not likely be uniformly distributed. More discussion on the use of eyewitness accounts in determining the prior probability is warranted.

While the above discussion focuses on the inaccuracy of eyewitness accounts, other assumptions that have been used to contribute to increasing the prior probability can be misleading and/or have been misused in missing persons identifications. These include location, open vs. closed properties, postmortem interval (PMI) and demographic variables often combined using the product rule. Law enforcement officers and other investigators (including some anthropologists) have based prior probabilities on assumptions about where the remains were found. For example, a decedent is deemed to be a member of a subset of a population of border crossers. People residing near borders can share a number of characteristics, and thus the candidate population may be greater. Prior probabilities should reflect the uncertainty of population affinity where assumptions about borders are inferred to reduce *v*. In addition, restricting a candidate population to the geographic region where the individual was last seen during a war does not reflect the possibility of transport of individuals into and out of that region.

The topic of closed and open populations has been addressed somewhat for mass disasters and does not need further discussion *per se*. However, other population factors should be considered. For example, anthropologists, odontologists and others use airplane manifests or other transportation manifests to establish 1/*v *and proceed to establish an antemortem database on the basis of the manifests. This database may not be accurate if some of the decedents falsified their identities, clearly a potential practice when the individuals are instrumental in the cause of the incident. Alternatively, consider how many "Mr Smiths" travel to Las Vegas every year. Databases need to be assessed and curated continuously.

PMI has been used to reduce 1/*v *in situations where a portion of the dead were killed within a particular time period. For example, 500 individuals may have been murdered during a one-year period and buried at a site, yet 5,000 people may have died over a ten-year period that overlaps the period of the mass murders. Separating the 500 individuals to set 1/*v *at 1/500 may not be defensible without substantial information that is often soft in such scenarios. PMIs are less reliable for intermediate-and long-term ranges than they are for recent time periods (hours or days) [35].

Demographic characteristics sometimes are used to reduce 1/*v*. Consider unidentified human remains found near a town of *N *inhabitants. An assumption might be made that 1/*v *is bounded by 1/*N*. Then attempts may be made to reduce 1/*v *by gender, age, stature, population affinity and so forth to increase the prior probability. Estimating the prior probability is not justified by applying the product rule of estimates of the assumed frequencies of the variables without supporting data from the geographic region. Not all demographic variables are independent [36,37], and they can be location-dependent (for example, gender at a women's prison is a nested variable).

Indeed, this practice of assuming independence of demographic characteristics is somewhat similar to what was proffered in the infamous *California v Collins *case in 1968 [38]. Collins and his wife were accused of a second-degree robbery, and to convey Collins's guilt, the prosecutor entered into evidence via an expert in mathematics some "demographic" data (Table 2). The product rule was applied to combine the factors, and a probability was derived that only 1 in 12 million couples would present these particular characteristics. It should be fairly obvious that these characteristics may not be independent. For example, it would seem plausible that a man with a beard would be more likely to have a mustache than these two characteristics being mutually independent (although that plausibility is an assumption that we have made as well). Upon appeal, the court overturned the conviction because the values for each characteristic were estimated without any supporting data and were assumed to be independent without any basis. Interestingly, the appellate court also raised the issue that the probability evidence did not factor into the reliability of an eyewitness account (as discussed above) and did not take into account other factors, such as a robber wearing a disguise. Clearly, the same principles of combining demographic information to raise the prior probability apply to missing persons identifications were used in the *Collins *case. It is disconcerting that 40 years after the *Collins *case, similar misuse of data to establish prior probabilities in missing persons cases may still be occurring.

**Table 2:** The characteristics and frequencies used in *California v Collins*^a^

Characteristic	Estimated frequency
Partly yellow automobile	1/10
Man with mustache	1/4
Girl with ponytail	1/10
Girl with blond hair	1/3
Negro man with beard	1/10
Interracial couple	1/1,000

^a^Data are derived from [38]. The combined probability under the assumption of independence that was presented in court was 1/12,000,000.

Lastly, the estimated age (mean age ± 1 standard deviation (SD)) has been used to reduce *v*. For example, the estimated age of the unidentified person could be reported as 50 ± 10 years based on examination of the morphological staging of the pubic symphyseal face. On the basis of this range, only individuals who were reported missing and were between the ages of 40 and 60 years were used to set the prior probability. Such an approach underestimates the number of individuals who should be included in establishing the prior probability. While there are several methods for estimating age and several data sets, for illustration purposes, we refer to the Suchey-Brooks method and data reported by Brooks and Suchey [39] and Katz and Suchey [40,41], who examined morphological changes of the pubic symphyseal region as an indicator of age at death. The data show that the range of uncertainty increases with the age of the remains. Estimation of age based on the morphology of the pubic symphyseal region is more reliable in individuals younger than 40 years of age (confirmed for Japanese, see [42]). Standard deviations increased notably for individuals over 45 years of age. Katz and Suchey [40,41] reported a mean age of 45.6 years with a 1 SD value of 10.4 years for males. This is similar to the value of 50 ± 10 years presented above. Yet, a range based on a ± 1 SD interval is not the same as the 95% confidence range, which is 27 to 66 years. While an age estimation of 50 ± 10 years is useful for prioritizing an investigation, it is not proper for eliminating all individuals younger than 40 and older than 60 years old. Prioritization for investigation does not translate equally to uncertainty regarding the age of remains. Also, there are suggestions that estimates would be more reliable if population affinity data were used [42,43]. Age estimates based on populations that do not represent the unidentified person also may carry more uncertainty. Likewise, similar uncertainties are relevant for estimating other anthropometric features on the basis of the skeletal remains of unidentified persons (for example, estimation of stature from skeletal remains [44]).

## Conclusion

A few examples have been provided of potential misuse of data to set a prior probability. More examples could have been provided, but the discussion herein is sufficient to make those involved in the identification of missing persons aware of the issues that may affect the establishment of prior probabilities. There should be agreement that it is not appropriate to assume 100% accuracy of an eyewitness account, that some demographic variables are not independent, that setting a prior probability at 1/*n *or assuming anthropological data are uniformly distributed may not be defensible and that it is necessary to have supporting data when invoking the frequency of occurrence of characteristics. Those involved in identifying human remains should be cognizant of these constraints and be cautious when establishing prior probabilities. These uncertainties should be considered when establishing identity, and officials (who are not scientifically adept) should be apprised of the limitations of prior probabilities so that processes can be carried out as reliably as possible. The human identity community should establish guidelines regarding objective formulations of prior odds.

## Authors' contributions

While all authors contributed equally to the manuscript, BB, JG and RC contributed more so to its genetic aspects, and BB and HGK contributed more so to its anthropologic aspects. All authors read and approved the final manuscript.
